# Supplementary material for: A selective enrichment and specific probe terminal mediated strategy for highly sensitive detection of microRNAs
Source: Nat Commun. 2026 Mar 16;17:4377. doi: 10.1038/s41467-026-70811-7 (PMC13184116; doi:10.1038/s41467-026-70811-7)
Supplement: Supplementary file 1 — Supplementary information [file 41467_2026_70811_MOESM1_ESM.pdf]

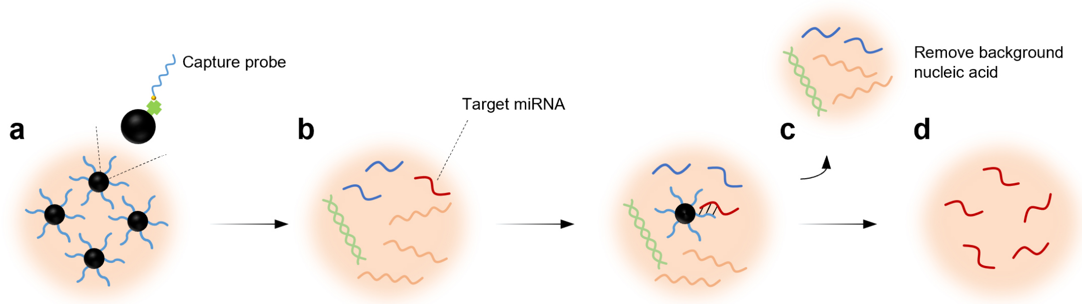

Supplementary Fig. 1. Major components and workflow of the selective enrichment method. **a** Superparamagnetic beads carry miRNA-specific capture probes with reverse complementary sequences. **b** miRNAs in biofluid samples are released and freely dispersed through lysis. **c** Functionalized magnetic beads selectively capture target miRNAs via base complementarity, while unbound nucleic acids are removed during washing. **d** Enriched miRNAs undergo high-temperature elution for downstream analysis.

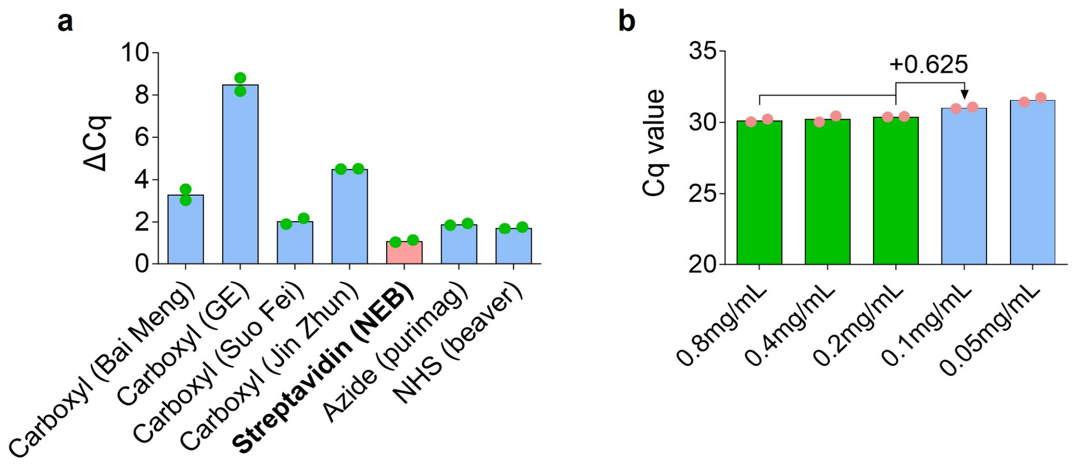

Supplementary Fig. 2. Screening of magnetic bead types and optimization of streptavidin magnetic bead concentration. a Comparison of miRNA capture efficiency among superparamagnetic beads functionalized with carboxyl, streptavidin, azide, or NHS groups. Capture probes (3′-amino-labeled for carboxyl/azide/NHS beads; 3′-biotin-labeled for streptavidin beads) are conjugated following manufacturer protocols. Streptavidin beads demonstrate superior miRNA capture performance under identical conditions. n = 2. **b** miRNA enrichment efficiency remains high across streptavidin bead concentrations of 0.2–0.8 mg/mL. n = 2. Cq, Quantification cycle.

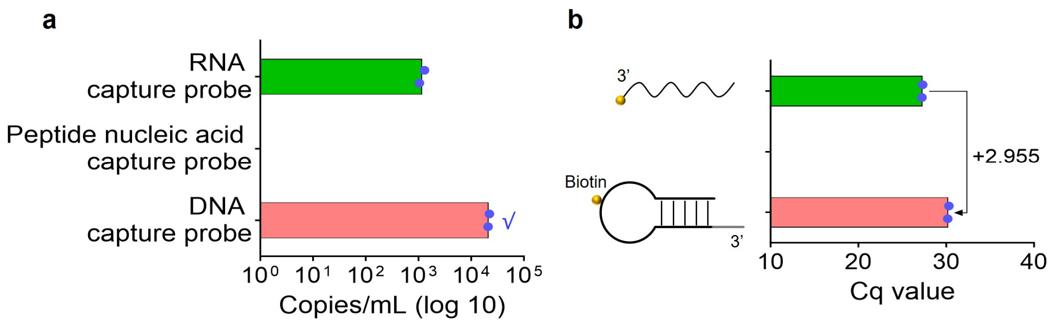

Supplementary Fig. 3. Screening of capture probe types and structures. a Comparison of miRNA enrichment efficiency among 3′-biotin-labeled RNA, PNA, and DNA capture probes. DNA probes demonstrate superior miRNA enrichment efficiency compared to RNA and PNA probes under identical conditions. n = 2. **b** Functional modification of stem-loop primers (previously used for reverse transcription) as miRNA capture probes. Linear probes exhibit higher miRNA enrichment efficiency than stem-loop structured probes. n = 2. Cq, Quantification cycle.

a

Name of the capture probe	Sequence	Modified
Capture probe-6nt	CGGTGA	3'Biotin
Capture probe-8nt	CCCGGTGA	3'Biotin
Capture probe-10nt	CACCCGGTGA	3'Biotin
Capture probe-12nt	TACACCCGGTGA	3'Biotin
Capture probe-14nt	TTTACACCCGGTGA	3'Biotin
Capture probe-16nt	GATTTACACCCGGTGA	3'Biotin

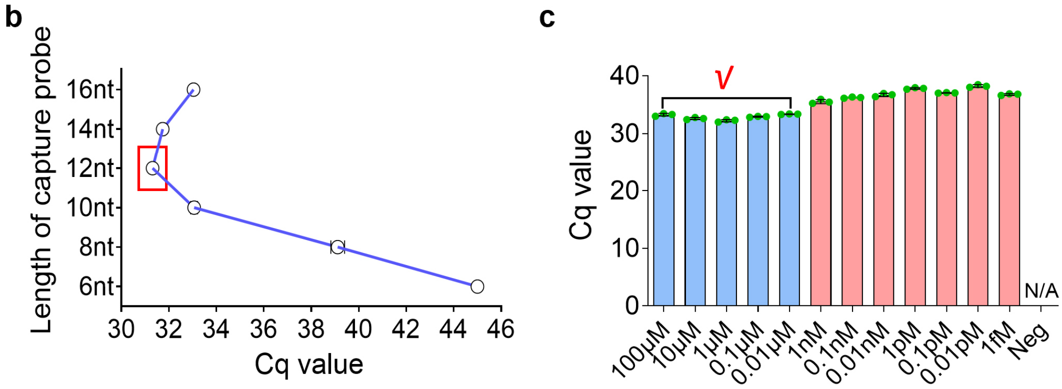

Supplementary Fig. 4. Optimization of capture probe length and coupling concentration. a miRNA enrichment efficiency evaluated with 5′-terminal incremental truncation of capture probes. **b** miRNA enrichment efficiency peaks at intermediate probe lengths and declines with further truncation. n = 3. **c** Probe coupling concentration optimization identifies 0.01 - 100 μM as the optimal range for maximal miRNA enrichment. n = 3. N/A, no amplification curve. M, mol/L. Cq, Quantification cycle.

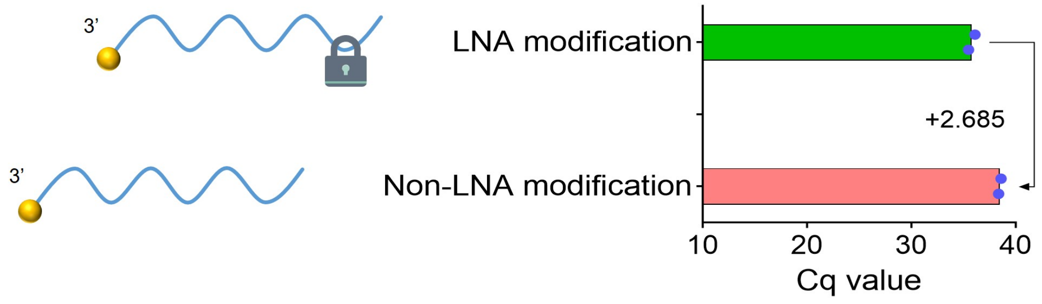

Supplementary Fig. 5. Effect of locked nucleic acid (LNA) modification on miRNA enrichment efficiency. LNA-modified capture probes enhance miRNA enrichment efficiency, achieving a ΔCq value of 2.685 compared to unmodified probes. The lock image designed by Freepik. n = 2. Cq, Quantification cycle.

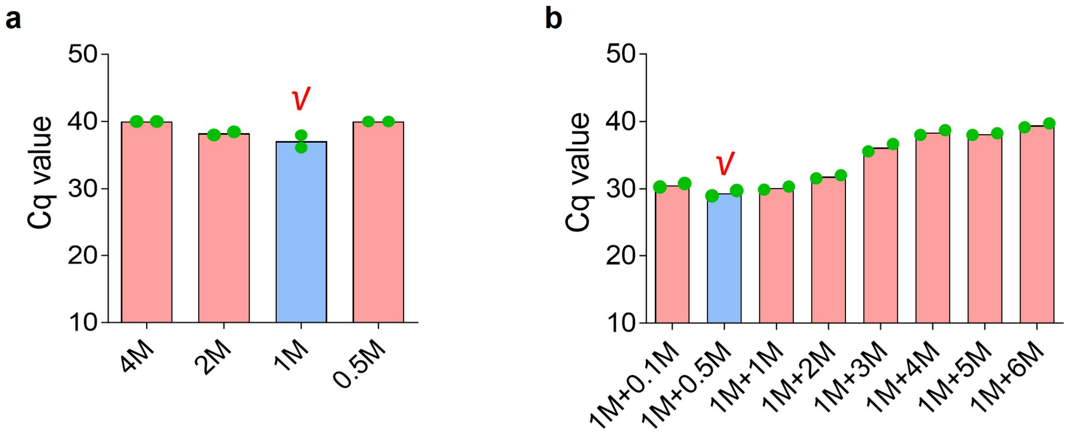

Supplementary Fig. 6. Screening and concentration optimization of lysis buffer. a Stepwise 2-fold dilution of 4 M guanidine isothiocyanate (GITC) for plasma lysis and miRNA extraction validation. Lysis efficiency peaks at 1 M GITC concentration. n = 2. **b** Synergistic lysis optimization by combining 1 M GITC with guanidine hydrochloride (GuHCl). The 0.5 M GuHCl and 1 M GITC hybrid system demonstrates enhanced plasma lysis efficiency and improved miRNA yield. n = 2. M, mol/L. Cq, Quantification cycle.

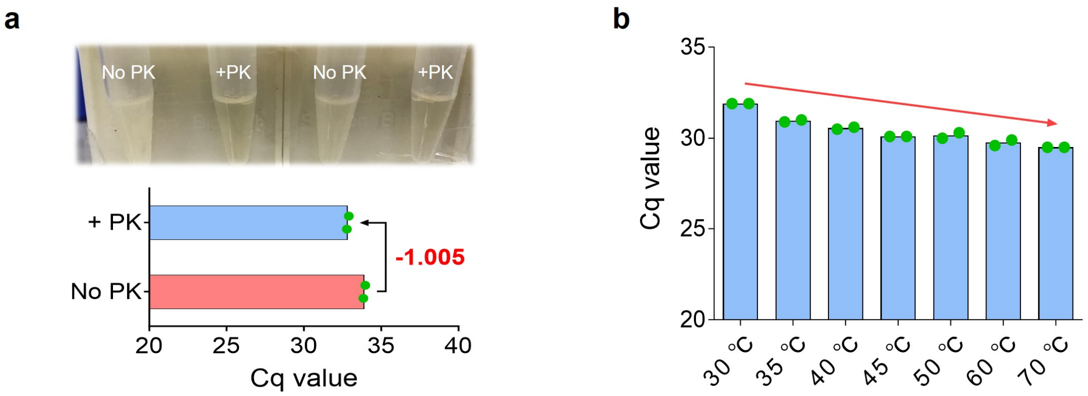

Supplementary Fig. 7. Validation of the hydrolysis efficiency of proteinase K and optimization of miRNA elution temperature. a Addition of 0.4 mg/mL proteinase K induces visible lysate clarification (milky white transformed ijnto yellowish-brown) and enhances miRNA recovery efficiency, exhibiting a ΔCq of -1 compared to untreated controls. n = 2. **b** Thermally assisted miRNA elution efficiency analysis. miRNA release efficiency positively correlates with temperature elevation, reaching maximal yield at 70°C. n = 2. PK, proteinase K. Cq, Quantification cycle.

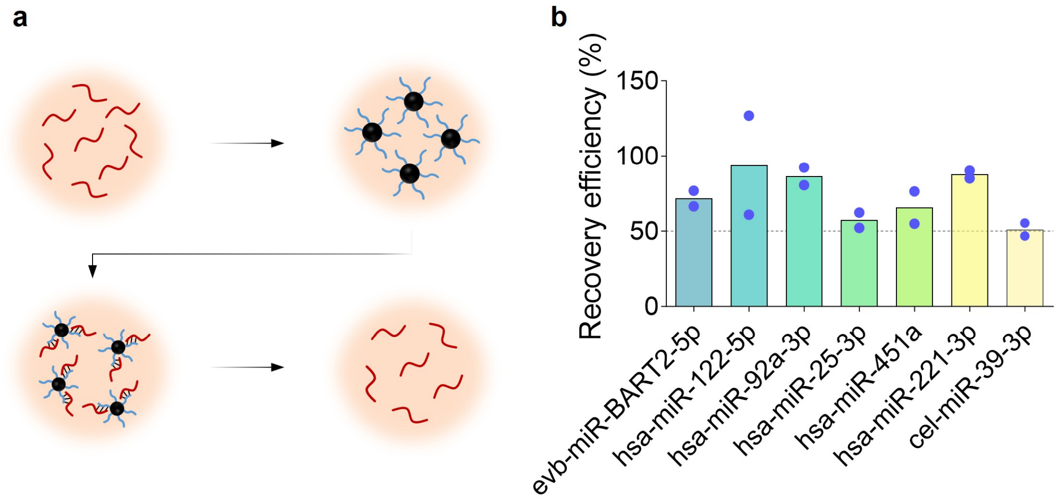

Supplementary Fig. 8. Recovery rate evaluation of automated selective enrichment method. a Recovery quantification protocol: Synthetic miRNAs with defined concentrations undergo selective enrichment processing, with yield calculation based on pre-/post-extraction content differential analysis. **b** Cross-species miRNA recovery efficiency: The selective enrichment system achieves >50% yield for pan-species miRNA targets (1 viral miRNA, 5 human miRNAs and 1 nematode miRNA), demonstrating broad-spectrum extraction capability. n = 2.

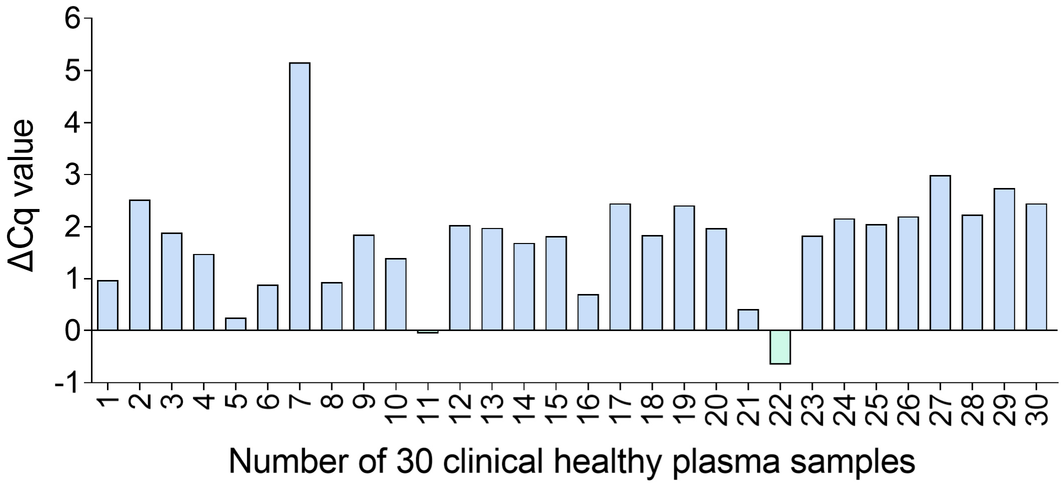

Supplementary Fig. 9. Performance comparison between automated selective enrichment and QIAGEN column-based method for human miRNA extraction in clinical plasma. The selective enrichment protocol demonstrates superior recovery efficiency versus the commercial QIAGEN kit (benchmarked as industry standard), achieving higher yields of endogenous hsa-miR-122-5p in >93% of clinically healthy donor samples. In the residual 7% cases, selective enrichment protocol maintains parity with QIAGEN's performance. Cq, Quantification cycle.

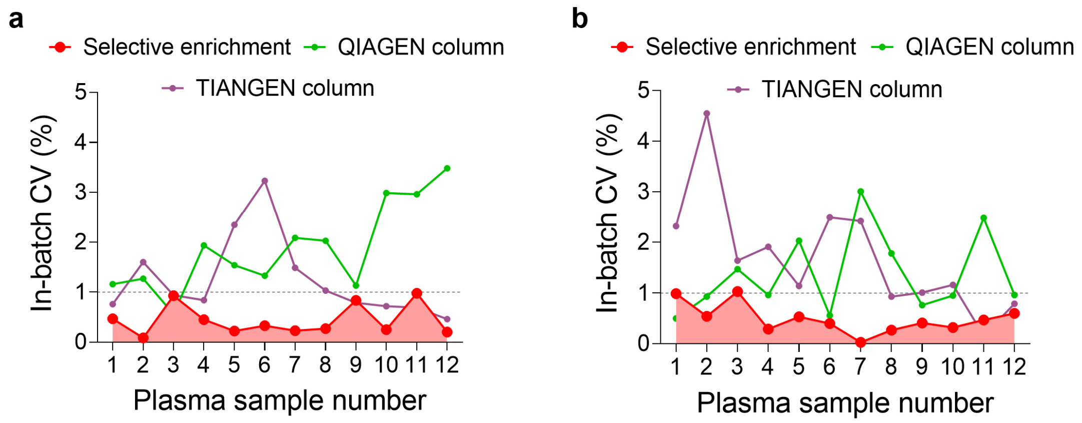

Supplementary Fig. 10. Intra-batch reproducibility assessment of exogenous and endogenous miRNA extraction in plasma. a Reproducibility of exogenous cel-miR-39-3p (spike-in control) quantification. n=3. **b** Reproducibility of endogenous hsa-miR-25-3p quantification. Cq values are analyzed with coefficient of variation (CV) values <1% for both targets, demonstrating high analytical precision. n=3.

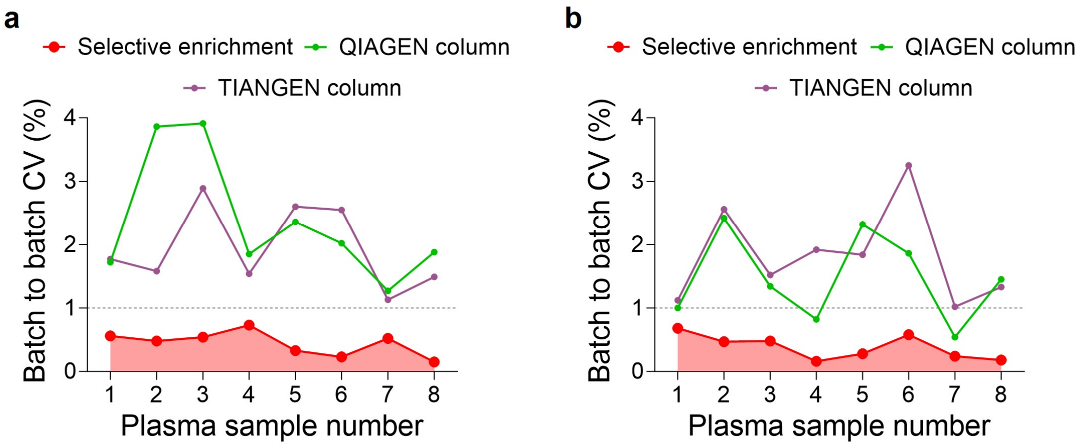

Supplementary Fig. 11. Inter-batch reproducibility evaluation of exogenous and endogenous miRNA extraction from plasma. **a** Inter-batch variation analysis of exogenous *C. elegans* cel-miR-39-3p (spike-in control) quantification. n=3. **b** Inter-batch variation analysis of endogenous hsa-miR-25-3p quantification. Cq values from three independent batches are analyzed with coefficient of variation (CV) values <1%, confirming robust inter- batch reproducibility. n=3.

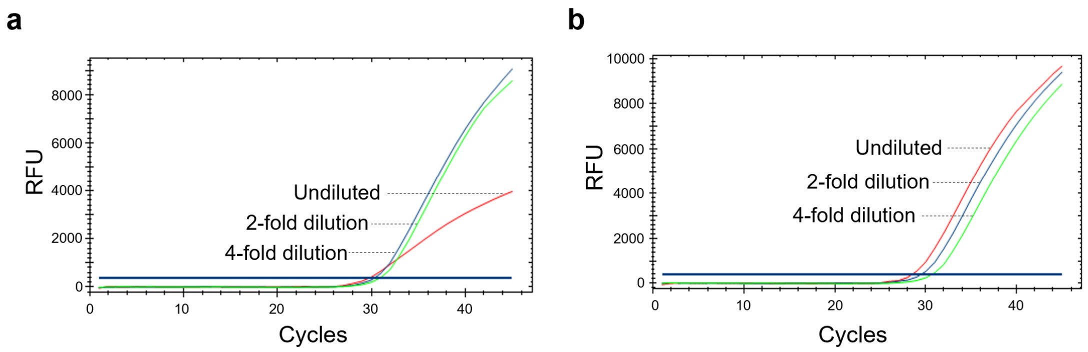

Supplementary Fig. 12. Detection results following extraction using both the QIAGEN column extraction method and the selective extraction method. a The detection results from the QIAGEN column extraction method. **b** The detection results from the selective extraction method. RFU, relative fluorescence units.

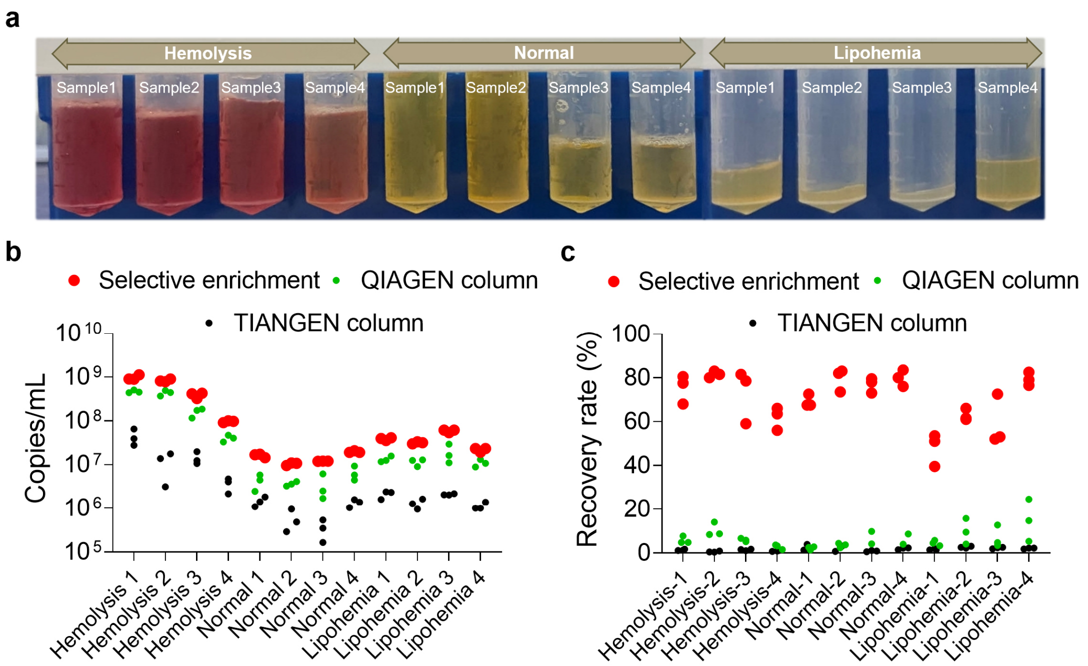

Supplementary Fig. 13. Detection of endogenous and exogenous miRNAs in various sample types using three extraction methods. a Plasma samples under hemolytic, normal, and lipemic conditions. **b** Endogenous miRNA levels in plasma samples extracted by the three methods. **c** Recovery rates of exogenous miRNA in plasma samples extracted by the three methods.

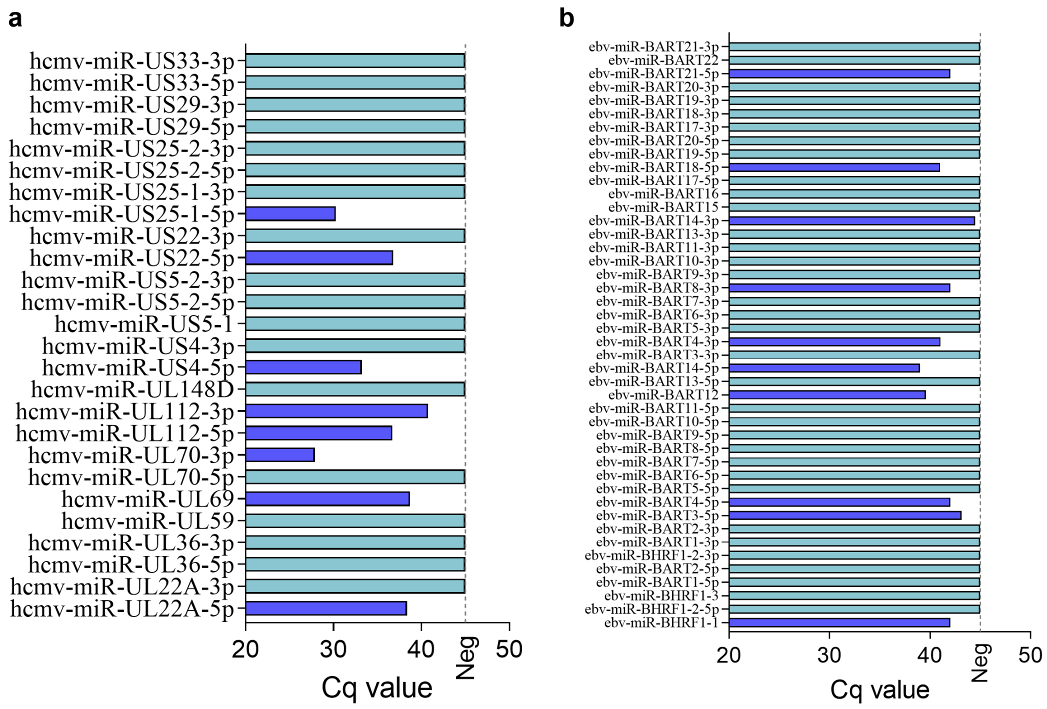

Supplementary Fig. 14. Specificity validation of the detection system for 26 HCMV miRNAs and 44 EBV miRNAs. a Detection results for 26 HCMV miRNAs. **b** Detection results for 44 EBV miRNAs. No template water (n=1). Neg, represents negative. Cq, Quantification cycle.

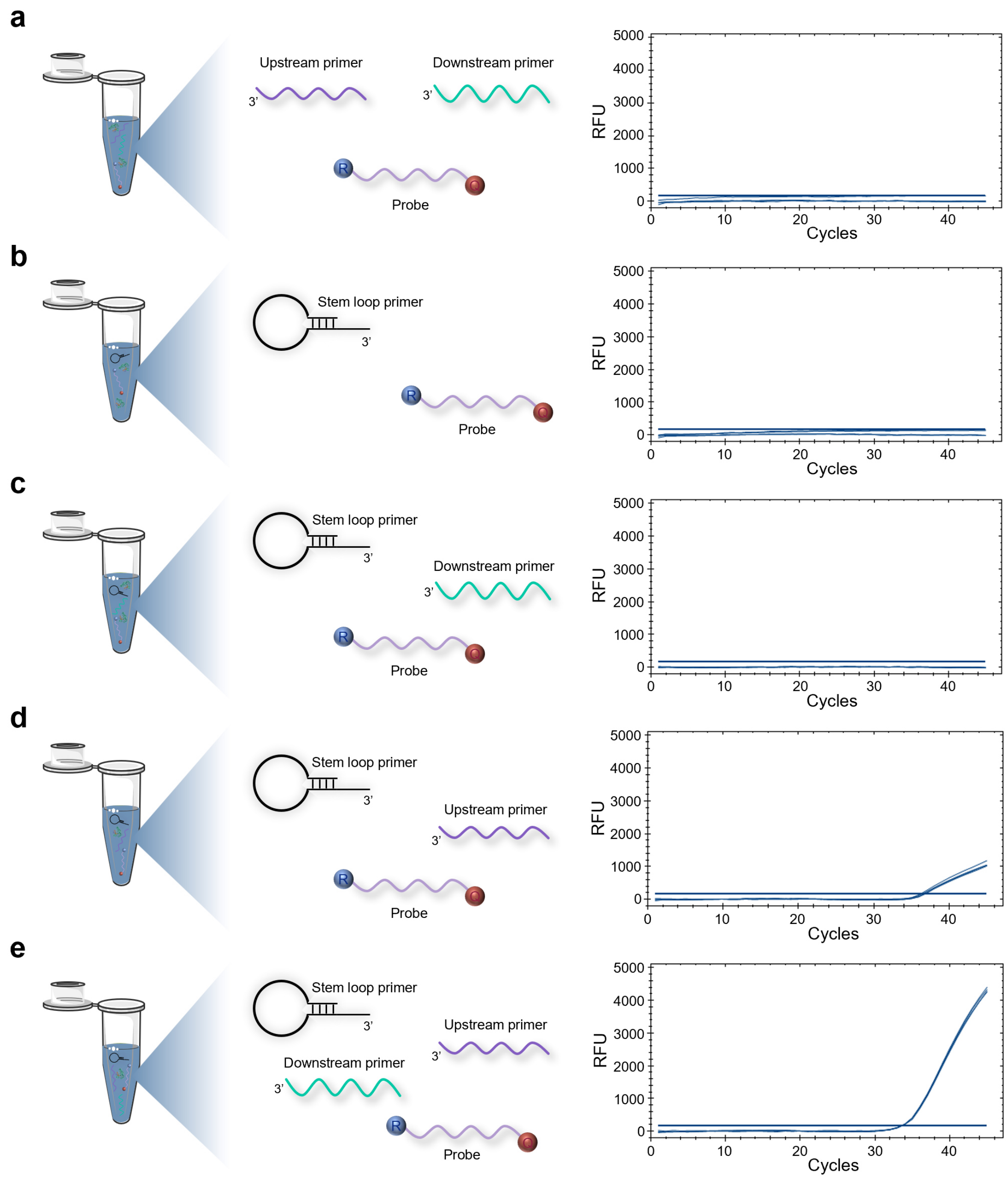

Supplementary Fig. 15. Investigation of non-specific amplification with different primer-probe combinations. a Combination of upstream primer, downstream primer, and TaqMan probe. **b** Combination of stem-loop primer and TaqMan probe. **c** Combination of stem-loop primer, downstream primer, and TaqMan probe. **d** Combination of stem-loop primer, upstream primer, and TaqMan probe. **e** Combination of stem-loop primer, upstream primer, downstream primer, and TaqMan probe. Free illustration materials are adapted from Bioicons. RFU, relative fluorescence units.

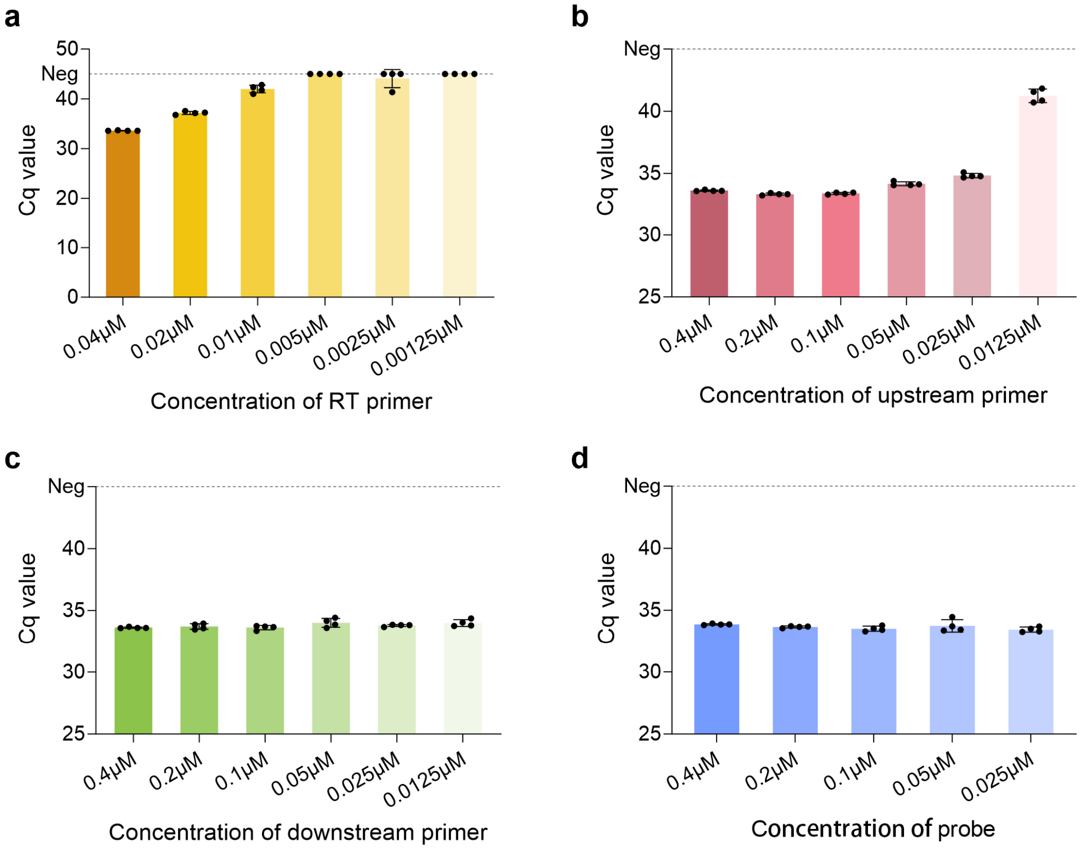

Supplementary Fig. 16. Effect of primers and probe concentrations on non-specific amplification. a Stem-loop primers in the range of 0.04μM-0.00125μM. n=4. **b** Upstream primers in the range of 0.4μM-0.0125μM. n=4. **c** Downstream primers in the range of 0.4μM-0.0125μM. n=4. **d** TaqMan probes in the range of 0.4μM-0.025μM. Neg represents negative. RT represents reverse transcription. n=4. Cq, Quantification cycle. The neg means negative.

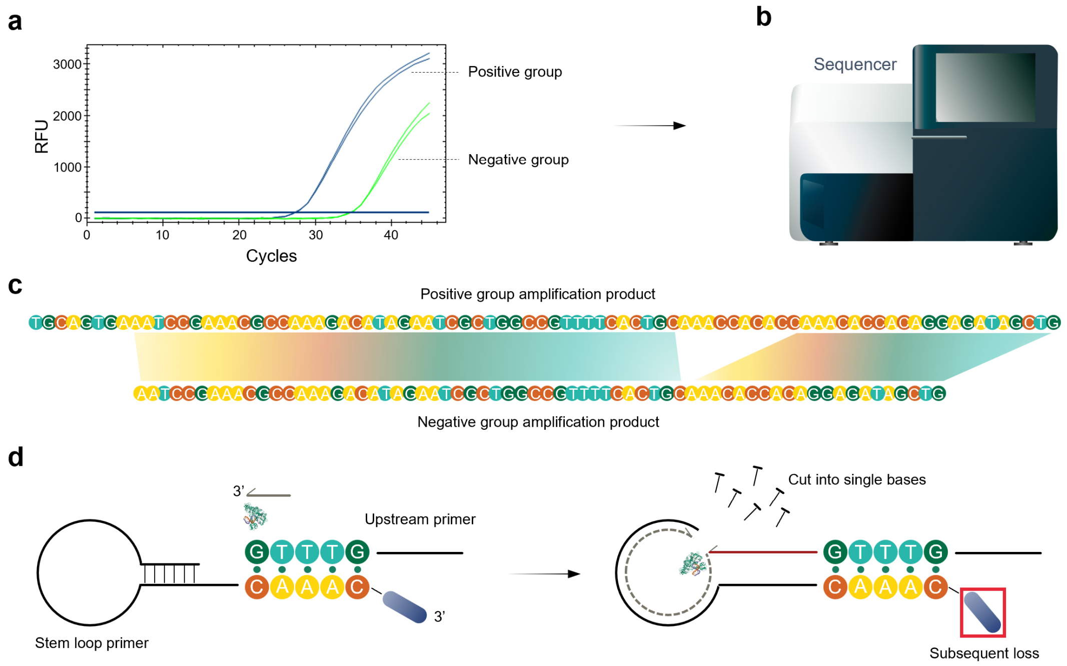

Supplementary Fig. 17. Analysis of non-specific amplification in stem-loop primer RT-qPCR. a Amplification curves of target miRNA (positive group) and no template control (negative group). **b** Next-generation sequencing (NGS) of amplified products from positive and negative reactions. Free illustration materials are adapted from Bioicons. **c** Sequence comparison of NGS results from positive and negative amplifications. **d** Sequence and structural analysis suggests that stem-loop and upstream primers may induce non-specific amplification via 3′-end partial base pairing during qPCR. Taq DNA polymerase (PDB: 5W6Q). Target: hsa-miR-BART12. RFU, relative fluorescence units.

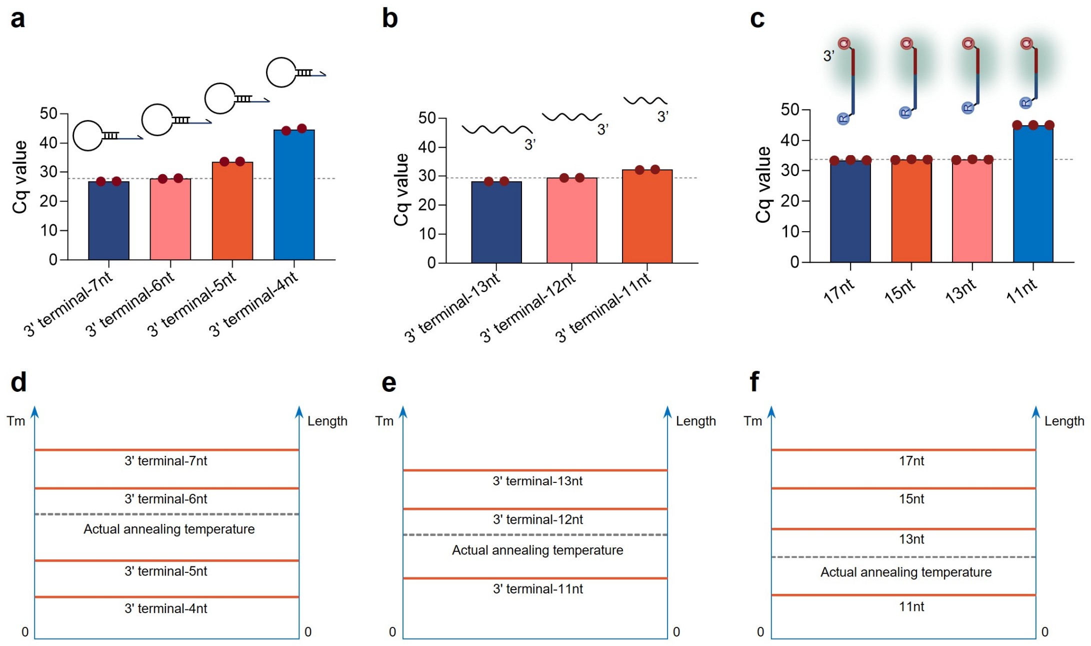

Supplementary Fig. 18. Development of specific probe terminal-mediated miRNA detection method. a and d Schematic diagrams of the reverse transcription efficiency and Tm values of stem-loop primers with different 3' terminal sequence lengths. n=2. **b and e** Schematic diagrams of the amplification efficiency and Tm values of upstream primers with different 3' terminal sequence lengths. n=2. **c and f** Schematic diagram of the amplification efficiency and Tm values of TaqMan probes with different sequence lengths. n=3. The nt means nucleotide. Cq, Quantification cycle. Tm, melting temperature. M, mol/L.

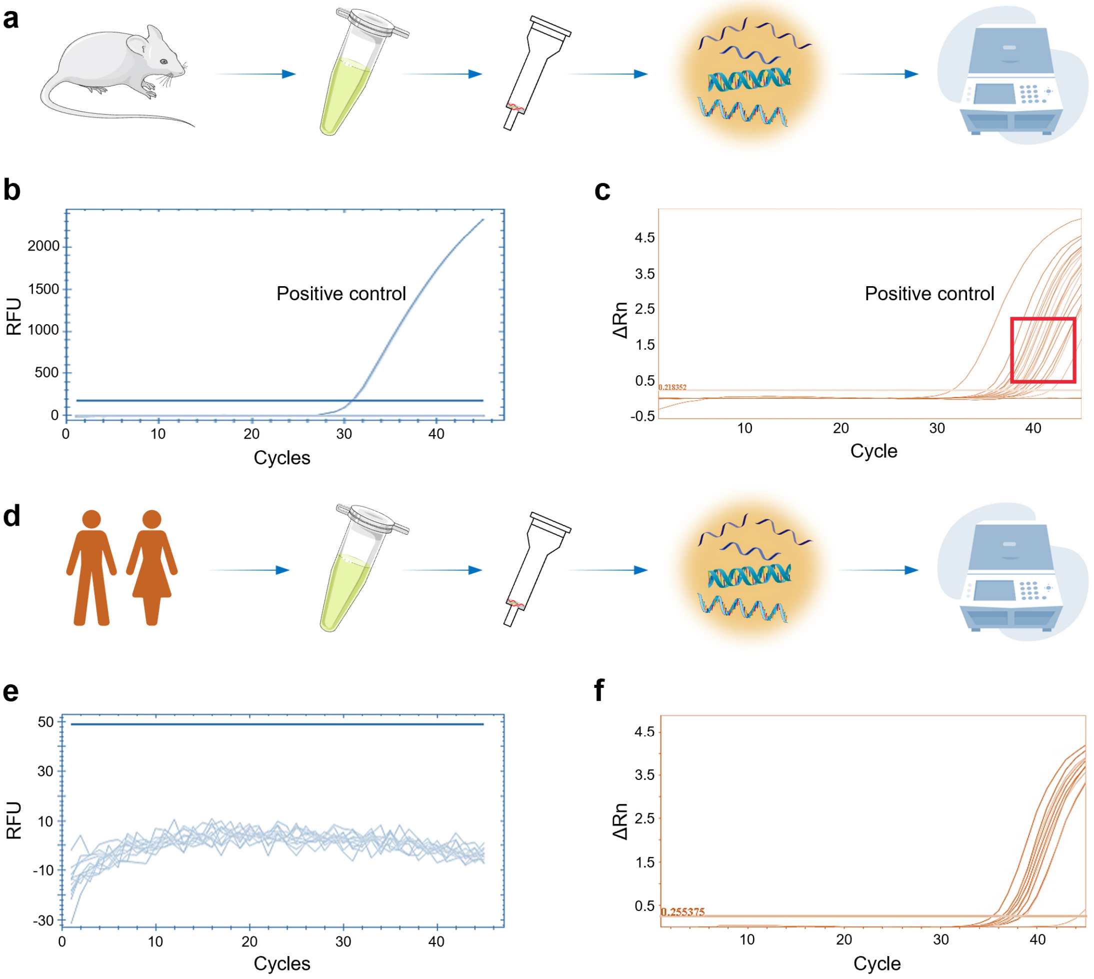

Supplementary Fig. 19. Specificity validation of different miRNA detection methods. a Schematic diagram of total RNA extraction and detection from healthy mouse plasma. Free illustration materials are adapted from Bioicons. **b** SPTM-PCR detection of nucleic acids from non-HCMV-infected mouse plasma (n=20 biologically independent samples). **c** Stem-loop RT-qPCR detection of nucleic acids from non-HCMV-infected mouse plasma (n=20 biologically independent samples). **d** Schematic diagram of total RNA extraction and detection from healthy human plasma. Free illustration materials are adapted from Bioicons and SciDraw. **e** SPTM-PCR detection of nucleic acids from non-HCMV-infected healthy human plasma (n=12 biologically independent samples). **f** Stem-loop RT-qPCR detection of nucleic acids from non-HCMV-infected healthy human plasma (n=12 biologically independent samples). Target: hcmv-miR-UL22A-5p. RFU, relative fluorescence units. ΔRn, delta normalized reporter.

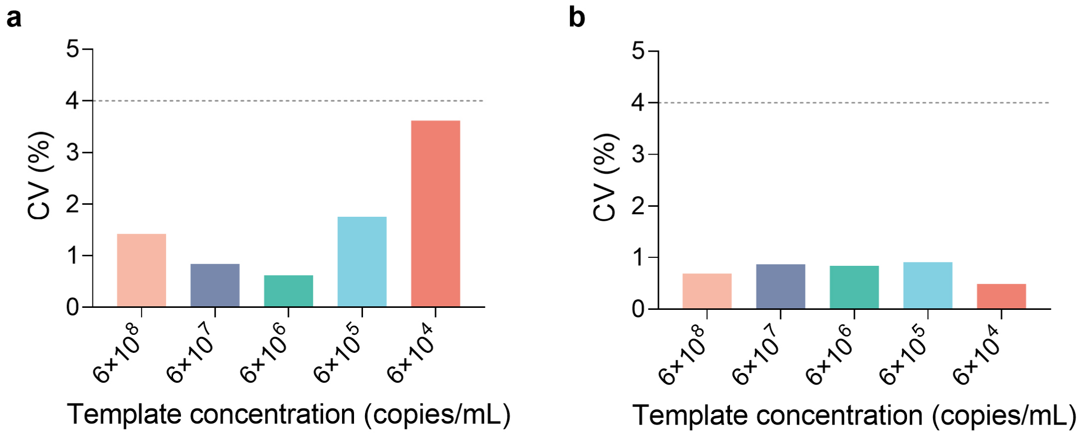

Supplementary Fig. 20. Reproducibility evaluation of the SPTM-PCR detection method. a Intra-batch reproducibility evaluation. n=12. **b** Inter-batch reproducibility evaluation. n=3. CV, coefficient of variation.

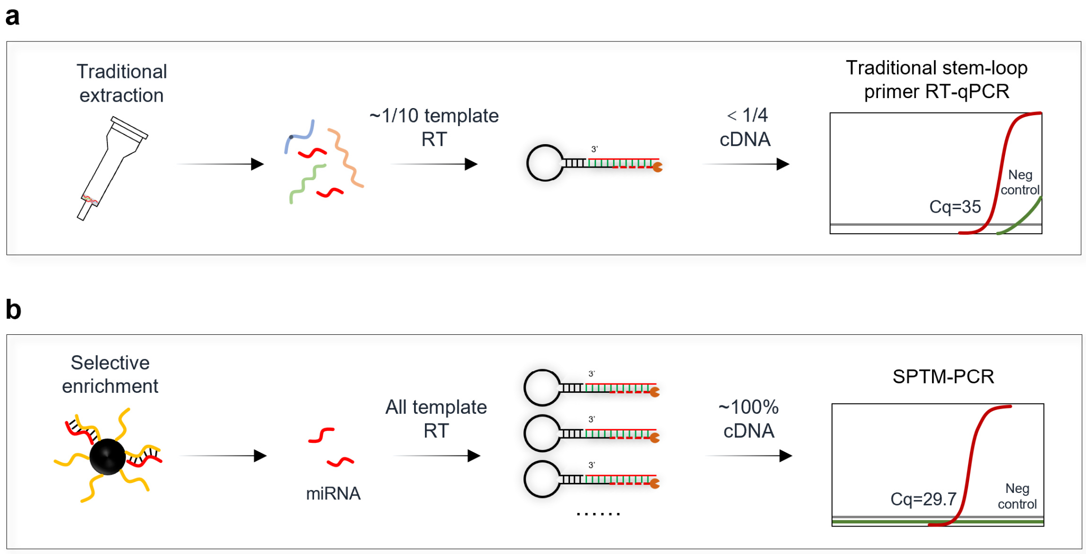

Supplementary Fig. 21. Comparison between traditional miRNA detection methods and the SE-SPTM-PCR high sensitivity miRNA detection method. a Traditional miRNA detection method. **b** High sensitivity miRNA detection method. The neg means negative. Cq, Quantification cycle. RT represents reverse transcription.

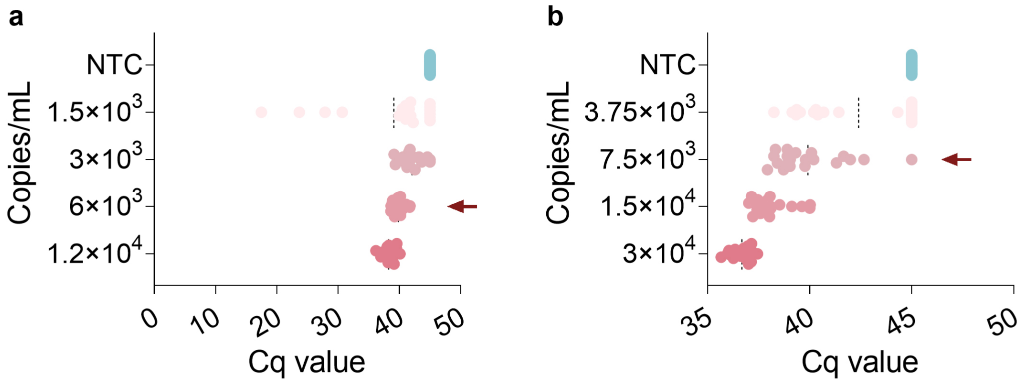

Supplementary Fig. 22. Evaluation of the lower detection limit of the SE-SPTM-PCR method using miRNA standards as templates. a hcmv-miR-22A-5p. n=20. **b** ebv-miR-BART2-5p. n=20. NTC, no templates water. Cq, Quantification cycle.

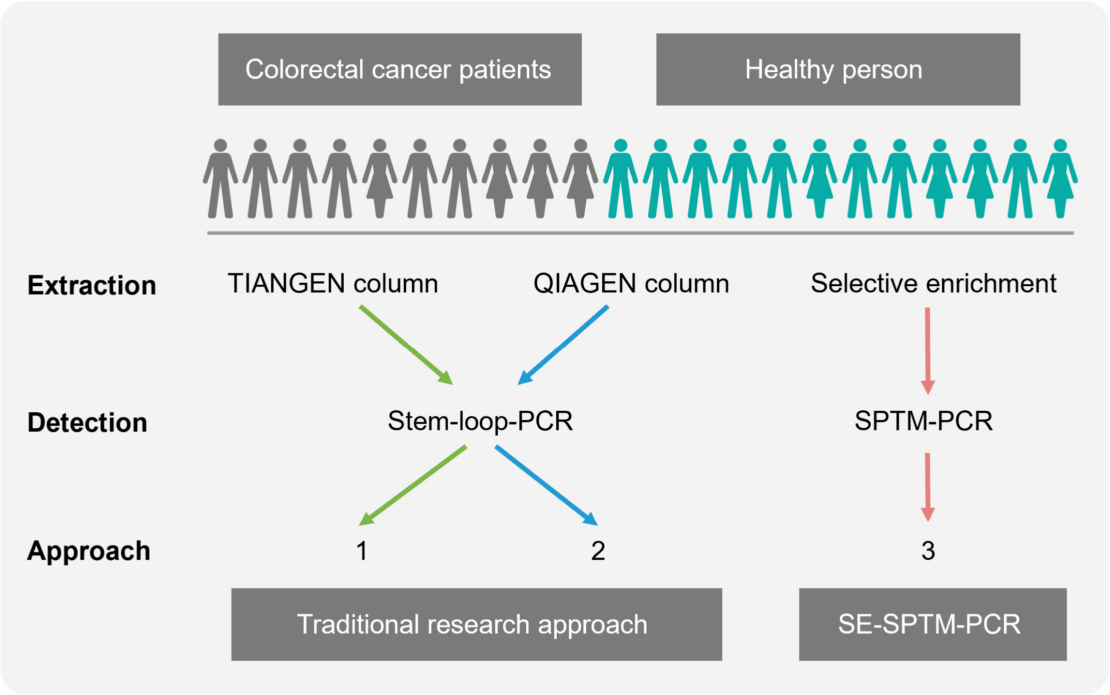

Supplementary Fig. 23. Detection of samples from colorectal cancer patients and healthy individuals using the traditional miRNA scheme and the SE-SPTM-PCR scheme. Colorectal cancer patients, n=48 biologically independent samples. Healthy individuals, n=48 biologically independent samples. Free illustration materials are adapted from SciDraw.

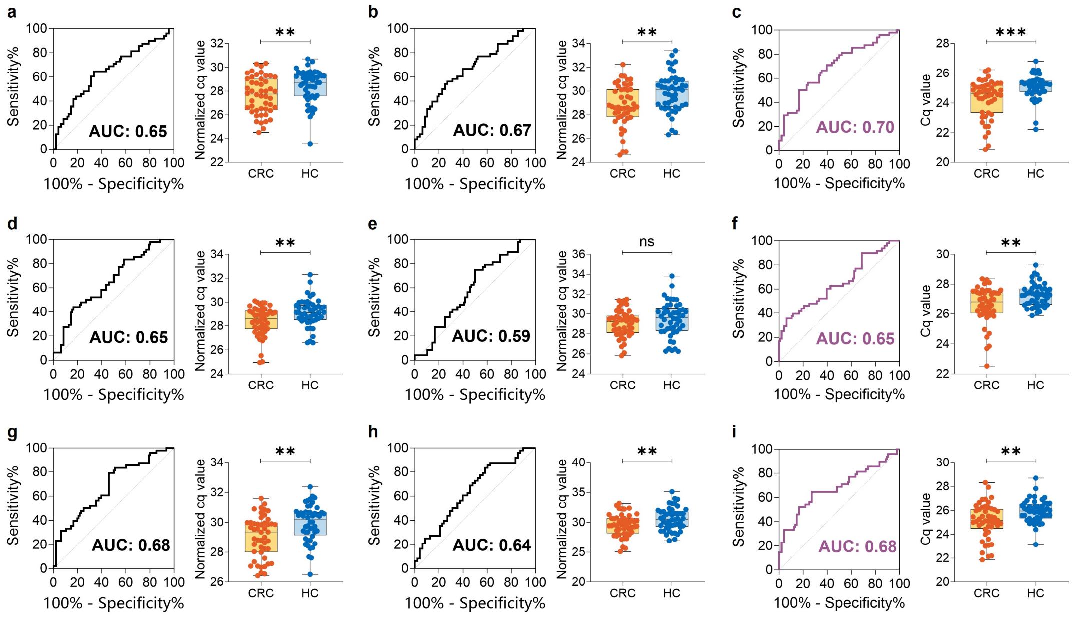

Supplementary Fig. 24. Diagnostic performance comparison of SE-SPTM-PCR versus conventional miRNA detection methods for CRC diagnosis using hsa-miR-320a (a-c), hsa-miR-19a-3p (d-f) and hsa-miR-423-5p (g-i). a, d, g Diagnostic performance of conventional scheme 1 (TIANGEN column extraction combined with stem-loop primer RT-qPCR). **b, e, h** Diagnostic performance of conventional scheme 2 (QIAGEN column extraction combined with stem-loop primer RT-qPCR). **c, f, i** Diagnostic accuracy of the SE-SPTM-PCR based detection system. CRC, colorectal cancer patients, n=48 biologically independent samples; HC, healthy controls, n=48 biologically independent samples. AUC, area under the curve. $^{**}P < 0.01$, $^{***}P < 0.001$, "ns" means not significant. The boxes indicate the interquartile range (IQR) of data between 75% (Q3) and 25% (Q1). The bars below and above each box indicate the data in Q1−1.5 × IQR and Q3+1.5 × IQR, respectively. Two-tailed Mann-Whitney U test.

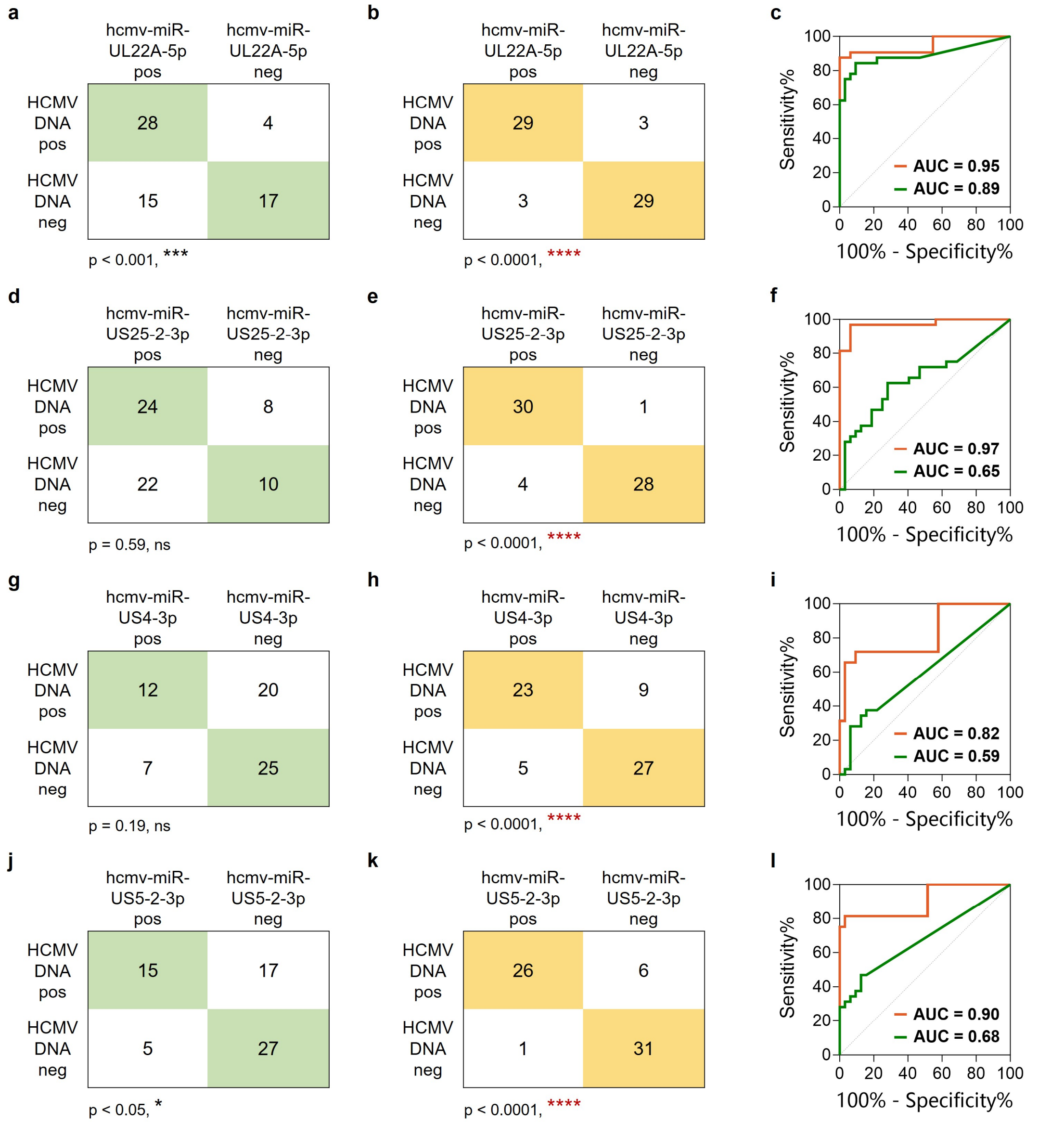

Supplementary Fig. 25. Diagnostic performance comparison of SE-SPTM-PCR and conventional miRNA detection system for monitoring HCMV reactivation in HSCT recipients. a, d, g, j Detection in HCMV DNA positive and negative HSCT patients using conventional RT-qPCR. Chi-square tests. **b, e, h, k** Detection in HCMV DNA positive and negative HSCT patients using SE-SPTM-PCR. Chi-square tests. **c, f, i, l** ROC curve analysis of hcmv-miR-UL22A-5p, hcmv-miR-US25-2-3p, hcmv-miR-US4-3p, hcmv-miR-US5-2-3p. The orange line represents SE-SPTM-PCR, and the green line represents conventional RT-qPCR. The pos means positive. The neg means negative. AUC, area under the curve.

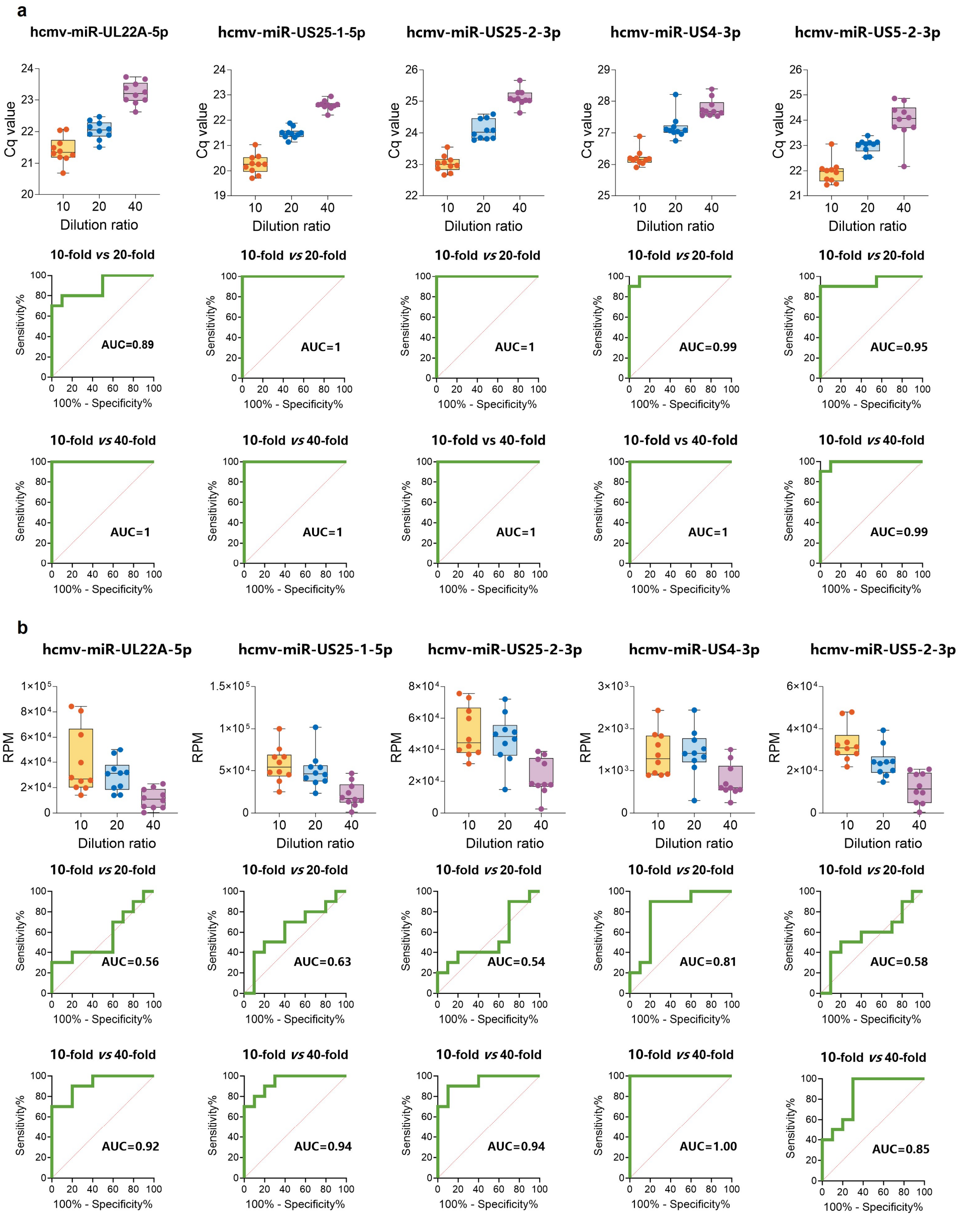

Supplementary Fig. 26. Diagnostic performance comparison of SE-SPTM-PCR and miRNA deep sequencing using 2-fold gradient dilutions of simulated HCMV viral infection samples. a The SE-SPTM-PCR method was used to detect HCMV miRNAs and the results were analyzed by ROC curve. **b** The deep sequencing method was used to detect HCMV miRNAs and the results were analyzed by ROC curve. AUC, area under the curve. The boxes indicate the interquartile range (IQR) of data between 75% (Q3) and 25% (Q1). The bars below and above each box indicate the data in Q1−1.5 × IQR and Q3+1.5 × IQR, respectively.

Supplementary Table 1 Reagent composition and reaction conditions for the entire miRNA selective enrichment process

	Lysis	Hybrid	Wash 1	Wash 2	Wash 3	Elution buffer
Components	1M Isothiocyanate guanidine, 0.5M Guanidine hydrochloride, 0.2mg Proteinase K	SA labeled magnetic bead, 10-15nt biotin-labeled DNA capture probe (100μM-0.01μM)	0.5M NaCl, 20mM Tris-HCl (PH=7.5), 1mM EDTA	0.5M NaCl, 20mM Tris-HCl (PH=7.5), 1mM EDTA	0.15M NaCl, 20mM Tris-HCl (PH=7.5), 1mM EDTA	10mM Tris-HCl (PH=7.5), 1mM EDTA
Reaction volume	100-500μL		100-500μL	100-500μL	100-500μL	100-500μL

M: mol/L. SA: streptavidin.

Supplementary Table 2 Automated selective enrichment parameters for miRNA

	Lysis and Hybrid	Wash 1	Wash 2	Wash 3	Elution buffer
Parameter	Moderate speed mixing 10min, Magnetic attraction 90s	Slow mixing 1min, Magnetic attraction 60s	Slow mixing 1min, Magnetic attraction 60s	No mixing required, Magnetic attraction 30s	Fast mixing 2min, Magnetic attraction 60s
Volume	100-500μL	100-500μL	100-500μL	100-500μL	25μL
Temperature	≤ 25℃	≤ 25℃	≤ 25℃	≤ 25℃	70℃

Supplementary Table 3 Comprehensive performance comparison between different extraction methods

Performance / Methods	Extraction time	Convenience degree	Cost/per person
Selective enrichment	20-25 min	Automation/ Manual	~4 CNY
QIAGEN column	~60 min	Automation/ Manual	~83 CNY
TIANGEN column	~80 min	Manual	~50 CNY
ABI non-specific magnetic bead	~90 min	Automation/ Manual	~54 CNY
Invitrogen Trizol	~60 min	Manual	~15 CNY

Supplementary Table 4 Artificial mutant sequences of hsa-miR-122-5p

Name	Sequence	Number of mutated bases
miR-122-reference	UGGAGUGUGACAAUGGUGUUUG	0
miR-122-mutation-1	UGGAGUGUGA**G**AAUGGUGUUUG	1
miR-122-mutation-2	UGGAGUGU**C**ACAAUGGUGUUUG	1
miR-122-mutation-3	UG**C**AG**A**GUGACAAUGGUGUUUG	2
miR-122-mutation-4	**A**GGAGUGU**C**ACAAUGGUGUUUG	2
miR-122-mutation-5	**A**G**C**AG**A**GUGACAAUGGUGUUUG	3

Bold bases indicate mutated bases.

Supplementary Table 5 Primers and probes for 26 HCMV-miRNA detection systems

Primer/probe name	Sequence	Modification
UL22A-5p-RT-1	TGCAGTGAAATCCGAAACGCCAAAGACACGAC TAGCTCCAAGCCATCACTGCA**TCTCACG**	/
UL22A-5p-F-1	TTCGTGACC**TAACTAGCCTTCC**	/
UL22A-3p-RT-1	TGCAGTGAAATCCGAAACGCCAAAGACACGAC TAGCTCCAAGCCATCACTGCA**CTACAAA**	/
UL22A-3p-F-1	AGGCG**TCACCAGAATGCTAG**	/
UL36-5p-RT-1	TGCAGTGAAATCCGAAACGCCAAAGACACGAC TAGCTCCAAGCCATCACTGCA**TCTTTCC**	/
UL36-5p-F-1	TCCGA**TCGTTGAAGACACCT**	/
UL36-3p-RT-1	TGCAGTGAAATCCGAAACGCCAAAGACACGAC TAGCTCCAAGCCATCACTGCA**GCACGTT**	/
UL36-3p-F-1	TCCGG**TTTCCAGGTGTTTTC**	/
UL59-RT-1	TGCAGTGAAATCCGAAACGCCAAAGACACGAC TAGCTCCAAGCCATCACTGCA**ACGGCAT**	/
UL59-F-1	TGGAAT**GTTCTCTCGCTCGTC**	/
UL69-RT-1	TGCAGTGAAATCCGAAACGCCAAAGACACGAC TAGCTCCAAGCCATCACTGCA**CGGTTTC**	/
UL69-F-1	TTGAAC**CCAGAGGCTAAGCC**	/
UL70-5p-RT-1	TGCAGTGAAATCCGAAACGCCAAAGACACGAC TAGCTCCAAGCCATCACTGCA**TCTGGAC**	/
UL70-5p-F-1	TGA**TGCGTCTCGGCCTC**	/
UL70-3p-RT-1	TGCAGTGAAATCCGAAACGCCAAAGACACGAC TAGCTCCAAGCCATCACTGCA**CCGCGCG**	/
UL70-3p-F-1	TTG**GGGGATGGGCTGG**	/
UL112-5p-RT-1	TGCAGTGAAATCCGAAACGCCAAAGACACGAC TAGCTCCAAGCCATCACTGCA**TGAGTAA**	/

UL112-5p-F-1	TAC**CCTCCGGATCACATGG**	/
UL112-3p-RT-1	TGCAGTGAAATCCGAAACGCCAAAGACACGAC<br>TAGCTCCAAGCCATCACTGCA**AGCCTGG**	/
UL112-3p-F-1	TAGGGC**AAGTGACGGTGAGAT**	/
UL148D-RT-1	TGCAGTGAAATCCGAAACGCCAAAGACACGAC<br>TAGCTCCAAGCCATCACTGCA**CGGTGAA**	
UL148D-F-1	TTGG**TCGTCCTCCCCTTC**	/
US4-5p-RT-1	TGCAGTGAAATCCGAAACGCCAAAGACACGAC<br>TAGCTCCAAGCCATCACTGCA**CAGACAT**	/
US4-5p-F-1	TG**TGGACGTGCAGGGGG**	/
US4-3p-RT-1	TGCAGTGAAATCCGAAACGCCAAAGACACGAC<br>TAGCTCCAAGCCATCACTGCA**AGAGGTG**	/
US4-3p-F-1	TTGGAA**TGACAGCCCGCTA**	/
US5-1-RT-1	TGCAGTGAAATCCGAAACGCCAAAGACACGAC<br>TAGCTCCAAGCCATCACTGCA**ACGCTCT**	/
US5-1-F-1	TTCCTG**TGACAAGCCTGACG**	/
US5-2-5p-RT-1	TGCAGTGAAATCCGAAACGCCAAAGACACGAC<br>TAGCTCCAAGCCATCACTGCA**CTTTCAG**	/
US5-2-5p-F-1	TTG**CTTTCGCCACACCTATC**	/
US5-2-3p-RT-1	TGCAGTGAAATCCGAAACGCCAAAGACACGAC<br>TAGCTCCAAGCCATCACTGCA**AGACATC**	/
US5-2-3p-F-1	TTGCGAAGT**TATGATAGGTGTGAC**	/
US22-5p-RT-1	TGCAGTGAAATCCGAAACGCCAAAGACACGAC<br>TAGCTCCAAGCCATCACTGCA**CCCGCGG**	/
US22-5p-F-1	TTGGAAG**TGTTTCAGCGTGTGT**	/
US22-3p-RT-1	TGCAGTGAAATCCGAAACGCCAAAGACACGAC<br>TAGCTCCAAGCCATCACTGCA**CCTGGTT**	/
US22-3p-F-1	**TCGCCGGCCGCGCTGT**	/

US25-1-5p-RT-1	TGCAGTGAAATCCGAAACGCCAAAGACACGAC TAGCTCCAAGCCATCACTGCA**GGTCCGA**	/
US25-1-5p-F-1	TTGC**AACCGCTCAGTGGC**	/
US25-1-3p-RT-1	TGCAGTGAAATCCGAAACGCCAAAGACACGAC TAGCTCCAAGCCATCACTGCA**GAGAACC**	/
US25-1-3p-F-1	TTGAC**TCCGAACGCTAGGTC**	/
US25-2-5p-RT-1	TGCAGTGAAATCCGAAACGCCAAAGACACGAC TAGCTCCAAGCCATCACTGCA**TCATCCA**	/
US25-2-5p-F-1	TTCG**AGCGGTCTGTTCAGG**	/
US25-2-3p-RT-1	TGCAGTGAAATCCGAAACGCCAAAGACACGAC TAGCTCCAAGCCATCACTGCA**CCGCGGG**	/
US25-2-3p-F-1	TTGC**ATCCACTTGGAGAGCT**	/
US29-5p-RT-1	TGCAGTGAAATCCGAAACGCCAAAGACACGAC TAGCTCCAAGCCATCACTGCA**CGTCACG**	/
US29-5p-F-1	TTGC**TGGATGTGCTCGGAC**	/
US29-3p-RT-1	TGCAGTGAAATCCGAAACGCCAAAGACACGAC TAGCTCCAAGCCATCACTGCA**TGATTGT**	/
US29-3p-F-1	AT**CCCACGGTCCGGGC**	/
US33-5p-RT-1	TGCAGTGAAATCCGAAACGCCAAAGACACGAC TAGCTCCAAGCCATCACTGCA**CGCCCAC**	/
US33-5p-F-1	TTG**GATTGTGCCCGGACC**	/
US33-3p-RT-1	TGCAGTGAAATCCGAAACGCCAAAGACACGAC TAGCTCCAAGCCATCACTGCA**TGGATGT**	/
US33-3p-F-1	TTGGT**TCACGGTCCGAGC**	/
Universal-R	AATCCGAAACGCCAAAGACA	/
Universal-P	CGACTAGCTCCAAGCCA	5’FAM, 3’MGB

Bold bases indicate miRNA related specific sequences. “/” means no modification. FAM: Carboxyfluorescein. MGB: minor groove binder.

Supplementary Table 6 Primers and probes for 44 EBV-miRNA detection systems

Primer/probe name	Sequence	Modification
BHRF1-1-RT-1	CCCTTGAGAATCCGAAACGCCAAAGACACGACTAGCTCCAAGCCACTCAAGGG**AACTCCG**	/
BHRF1-1-F-1	TAGTTCCA**TAACCTGATCAGCCC**	/
BHRF1-2-5p-RT-1	CCCTTGAGAATCCGAAACGCCAAAGACACGACTAGCTCCAAGCCACTCAAGGG**GCTATCT**	/
BHRF1-2-5p-F-1	CTCTGTCT**AAATTCTGTTGCAGC**	/
BHRF1-3-RT-1	CTCTGACCTGAATCCGAAACGCCAAAGACACGACTAGCTCCAAGCCACAGGTCAGAG**TGTGCTT**	/
BHRF1-3-F-1	CTCTGACA**TAACGGGAAGTGTGT**	/
BART1-5p-RT-1	CCCTTGAGAATCCGAAACGCCAAAGACACGACTAGCTCCAAGCCACTCAAGGG**CACAGCA**	/
BART1-5p-F-1	GTGCA**TCTTAGTGGAAGTGACG**	/
BART2-5p-RT-1	TGCAGTGAAATCCGAAACGCCAAAGACACGACTAGCTCCAAGCCATCACTGCA**GCAAGGG**	/
BART2-5p-F-1	CCGTACTC**TATTTTCTGCATTCG**	/
BHRF1-2-3p-RT-1	CTCTGACCTGAATCCGAAACGCCAAAGACACGACTAGCTCCAAGCCACAGGTCAGAG**TCAATTT**	/
BHRF1-2-3p-F-1	ATGTAGTA**TATCTTTTGCGGCAG**	/
BART1-3p-RT-1	TGCAGTGAAATCCGAAACGCCAAAGACACGACTAGCTCCAAGCCATCACTGCA**GACATAG**	/
BART1-3p-F-1	ATGTACA**TAAGCACCGCTATCCA**	/

BART2-3p-RT-1	CTCTGACCTGAATCCGAAACGCCAAAGACACGACTAGCTCCAAGCCACAGGTCAGAG**TTTATTT**	/
BART2-3p-F-1	GTAGTT**AAGGAGCGATTTGGAGA**	/
BART3-5p-RT-1	TGCAGTGAAATCCGAAACGCCAAAGACACGACTAGCTCCAAGCCATCACTGCA**AGCACAAC**	/
BART3-5p-F-1	GCGCG**ACCTAGTGTTAGTGTT**	/
BART4-5p-RT-1	CTCTGACCTGAATCCGAAACGCCAAAGACACGACTAGCTCCAAGCCACAGGTCAGAG**AGCACAC**	/
BART4-5p-F-1	ATGTACAA**GACCTGATGCTGCTG**	/
BART5-5p-RT-1	TGCAGTGAAATCCGAAACGCCAAAGACACGACTAGCTCCAAGCCATCACTGCA**CGATGGGCA**	
BART5-5p-F-1	TAGCTCCA**CAAGGTGAATATAGC**	/
BART6-5p-RT-1	TGCAGTGAAATCCGAAACGCCAAAGACACGACTAGCTCCAAGCCATCACTGCA**CCTATGG**	/
BART6-5p-F-1	TAGCTGTA**TAAGGTTGGTCCAAT**	/
BART7-5p-RT-1	CCCTTGAGAATCCGAAACGCCAAAGACATAGAATCGCTGGCCGTTTCTCAAGGG**TGTTTCATA**	/
BART7-5p-F-1	TGCG**CCTGGACCTTGACT**	/
BART8-5p-RT-1	CTCTGACCTGAATCCGAAACGCCAAAGACACGACTAGCTCCAAGCCACAGGTCAGAG**CTGTACA**	/
BART8-5p-F-1	GAGCTCAC**TACGGTTTCCTAGAT**	/

BART9-5p-RT-1	GGAAGACCGAATCCGAAACGCCAAAGACACGACTAGCTCCAAGCCACGGTCTTCC**GTTTCCA**	/
BART9-5p-F-1	AGCTCAC**TACTGGACCCTGAAT**	/
BART10-5p-RT-1	TGCAGTGAAATCCGAAACGCCAAAGACACGACTAGCTCCAAGCCATCACTGCA**TGTACAG**	/
BART10-5p-F-1	TCTATCT**GCCACCTCTTTGGTT**	/
BART11-5p-RT-1	TGCAGTGAAATCCGAAACGCCAAAGACACGACTAGCTCCAAGCCATCACTGCA**CAACTAGCG**	/
BART11-5p-F-1	AGCTCAC**TCAGACAGTTTGGTG**	/
BART12-RT-1	TGCAGTGAAATCCGAAACGCCAAAGACATAGAATCGCTGGCCGTTTTCACTGCA**AACCACAC**	/
BART12-F-1	CAGCTATC**TCCTGTGGTGTTTG**	/
Universal-P-1	TAGAATCGCTGGCCGTTT	5’FAM,3’ MGB
BART13-5p-RT-1	CCCTTGAGAATCCGAAACGCCAAAGACATAGAATCGCTGGCCGTTTCTCAAGGG**CTGTACG**	/
BART13-5p-F-1	TG**AACCGGCTCGTGGCT**	/
BART14-5p-RT-1	GGAAGACCGAATCCGAAACGCCAAAGACACGACTAGCTCCAAGCCACGGTCTTCC**TGTAAAT**	/
BART14-5p-F-1	ATATGTA**TACCCTACGCTGCCG**	/
BART3-3p-RT-1	TGCAGTGAAATCCGAAACGCCAAAGACACGACTAGCTCCAAGCCATCACTGCA**ACACCTG**	/
BART3-3p-F-1	AAGCG**CGCACCACTAGTCAC**	/

BART4-3p-RT-1	TGCAGTGAAATCCGAAACGCCAAAGACACGACTAGCTCCAAGCCATCACTGCA**ACACCTGG**	/
BART4-3p-F-1	TATCTAAC**CACATCACGTAGGCA**	/
BART5-3p-RT-1	CTCTGACCTGAATCCGAAACGCCAAAGACACGACTAGCTCCAAGCCACAGGTCAGAG**AGGTGAAC**	/
BART5-3p-F-1	TAATCTAAC**GTGGGCCGCT**	/
BART6-3p-RT-1	CTCTGACCTGAATCCGAAACGCCAAAGACACGACTAGCTCCAAGCCACAGGTCAGAG**TCTAAGGCT**	/
BART6-3p-F-1	ATCTAAT**CGGGGATCGGACTA**	/
BART7-3p-RT-1	AACCTGCACAATCCGAAACGCCAAAGACACGACTAGCTCCAAGCCAGTGCAGGTT**CCCTGGA**	/
BART7-3p-F-1	ATCGG**CATCATAGTCCAGTGT**	/
BART8-3p-RT-1	TGCAGTGAAATCCGAAACGCCAAAGACATAGAATCGCTGGCCGTTTTCACTGCA**TCTACGAC**	/
BART8-3p-F-1	GCTACTC**GTCACAATCTATGGG**	/
Universal-P-1	TAGAATCGCTGGCCGTTT	5’FAM, 3’MGB
BART9-3p-RT-1	TGCAGTGAAATCCGAAACGCCAAAGACACGACTAGCTCCAAGCCATCACTGCA**ACTACGGG**	/
BART9-3p-F-1	CCGTACTC**TAACACTTCATGGGT**	/
BART10-3p-RT-1	TGCAGTGAAATCCGAAACGCCAAAGACACGACTAGCTCCAAGCCATCACTGCA**ACAGCCAA**	/

BART10-3p-F-1	CCGTACTC**TACATAACCATGGAG**	/
	CTCTGACCTGAATCCGAAACGCCAAAGACA	
BART11-3p-RT-1	TAGAATCGCTGGCCGTTTCAGGTCAGAG**GG**	/
	**CAGTCAGC**	
BART11-3p-F-1	TCACTAAC**ACGCACACCAGG**	/
	AACCTGCACAATCCGAAACGCCAAAGACAC	
BART13-3p-RT-2	GACTAGCTCCAAGCCAGTGCAGGTT**TCAGC**	/
	**CG**	
BART13-3p-F-2	GGG**TGTAACTTGCCAGGGA**	/
	CTCTGACCTGAATCCGAAACGCCAAAGACA	
BART14-3p-RT-1	CGACTAGCTCCAAGCCACAGGTCAGAG**ATC**	/
	**CCTACT**	
BART14-3p-F-1	CCGATCTC**TAAATGCTGCAGT**	/
	CTCTGACCTGAATCCGAAACGCCAAAGACA	
BART15-RT-1	CGACTAGCTCCAAGCCACAGGTCAGAG**TCA**	/
	**AGGA**	
BART15-F-1	CGTACTC**GTCAGTGGTTTTGTT**	/
	CTCTGACCTGAATCCGAAACGCCAAAGACA	
BART16-RT-1	CGACTAGCTCCAAGCCACAGGTCAGAG**AGA**	/
	**GCAC**	
BART16-F-1	CGATGAC**TTAGATAGAGTGGGTGT**	/
	GGAAGACCGAATCCGAAACGCCAAAGACA	
BART17-5p-RT-1	CGACTAGCTCCAAGCCACGGTCTTCC**CTTG**	/
	**TAT**	
BART17-5p-F-1	TATCTAAC**TAAGAGGACGCAGGC**	/
	CTCTGACCTGAATCCGAAACGCCAAAGACA	
BART18-5p-RT-1	CGACTAGCTCCAAGCCACAGGTCAGAG**TGT**	/
	**ATAG**	

BART18-5p-F-1	AGATGAC**TCAAGTTCGCACTTC**	/
BART19-5p-RT-1	TGCAGTGAAATCCGAAACGCCAAAGACACGACTAGCTCCAAGCCATCACTGCA**CATGTCAT**	/
BART19-5p-F-1	ATATTAA**ACATTCCCCGCAAAC**	/
BART20-5p-RT-1	GGAAGACCGAATCCGAAACGCCAAAGACACGACTAGCTCCAAGCCACGGTCTTCC**GGAATGAAGA**	/
BART20-5p-F-1	CACGATCTC**TAGCAGGCATGT**	/
BART17-3p-RT-1	TGCAGTGAAATCCGAAACGCCAAAGACACGACTAGCTCCAAGCCATCACTGCA**ACTAAGGGGA**	/
BART17-3p-F-1	ACTGATCTC**TGTATGCCTGGTG**	/
BART18-3p-RT-1	CTCTGACCTGAATCCGAAACGCCAAAGACACGACTAGCTCCAAGCCACAGGTCAGAG**GACGAAGCC**	/
BART18-3p-F-1	TATCTC**TATCGGAAGTTTGGGC**	/
BART19-3p-RT-1	CTCTGACCTGAATCCGAAACGCCAAAGACACCTACCGGACGTCCCTTTCAGGTCAGAG**AGCATTC**	/
BART19-3p-F-1	CTGATCTC**TTTTGTTTGCTTGG**	/
BART20-3p-RT-1	GGAAGACCGAATCCGAAACGCCAAAGACACGACTAGCTCCAAGCCACGGTCTTCC**GGTAACAGG**	/
BART20-3p-F-1	ATCTTAA**CATGAAGGCACAGCC**	/
BART21-5p-RT-1	GGAAGACCGAATCCGAAACGCCAAAGACACGACTAGCTCCAAGCCACGGTCTTCC**GTTAGTT**	/
BART21-5p-F-1	ACTGAGCTC**TCACTAGTGAAGGC**	/

BART22-RT-1	CCCTTGAGAATCCGAAACGCCAAAGACACG ACTAGCTCCAAGCCACTCAAGGG**ACTACTA**	/
BART22-F-1	CTGAGCTC**TTACAAAGTCATGGTC**	/
BART21-3p-RT-1	CCCTTGAGAATCCGAAACGCCAAAGACACG ACTAGCTCCAAGCCACTCAAGGG**AAACACC**	/
BART21-3p-F-1	TGAAGTC**CTAGTTGTGCCCACT**	/
Universal-R	AATCCGAAACGCCAAAGACA	/
Universal-P-2	CGACTAGCTCCAAGCCA	5'FAM, 3'MGB

"RT" means reverse transcription primer. Bold bases indicate miRNA related specific sequences. "/" means no modification. "F" means forward primer. FAM: carboxyfluorescein. MGB: minor groove binder. "R" means reverse primer. "P" means probe.

Supplementary Table 7 Sequence information of the SE-SPTM-PCR detection systems

miRNA	Name	Sequence	Modification
hsa-miR-92a-3p	Capture probe	**GACAAGTGCAATA**	3'-biotin-TEG
	RT	GATAAACGTATCGCGTCTAGGTTCGAACTTCGTTTATC**ACAGGC**	/
	F	AGACACTGACTACGTA**TATTGCACTT**	/
	R	GTATCGCGTCTAGGTTCGAAC	/
	P	AGGC**CGGGAC**	5'FAM, 3'BHQ1
hsa-miR-320a	Capture probe	**ACCCAGCTTTT**	3'-biotin-TEG
	RT	GATAAACGTTCTTGAGTCTAGCATCGAGCATACGTTTATC**TCGCCC**	/
	F	ACGTATCT**AAAAGCTGGGTTG**	/
	R	TCTTGAGTCTAGCATCGAGCAT	/
	P	TC**TCGCCCTCT**	5'FAM, 3'BHQ1
hsa-miR-19a-3p	Capture probe	**GCATAGATTTGCACA**	3'-biotin-TEG
	RT	GATAAACGTTCTTGAGTCTAGCATCGAGCATACGTTTATC**TCAGT**	/
	F	TGACACG**TGTGCAAATCTATG**	/
	R	TCTTGAGTCTAGCATCGAGCAT	/

	P	CGTTTATC**TCAGTTTTG**	5’FAM, 3’BHQ1
hsa-miR-423-5p	Capture probe	**CTGCCCCTCA**	3’-biotin-TEG
	RT	GATAAACGTTCTTGAGTCTAGCATCGAGCATACGTTTATC**AAAGTCTC**	/
	F	CTTATCGAG**TGAGGGGCAGA**	/
	R	TCTTGAGTCTAGCATCGAGCAT	/
	P	C**AAAGTCTCGCTC**	5’FAM, 3’BHQ1
hcmv-miR-US25-1-5p	Capture probe	**CACTGAGCGGTT**	3’-biotin-TEG
	RT	GATAAACGTATCGCGTCTAGGTTCGAACTTCGTTTATC**GGTCC**	/
	F	CCATACGATT**AACCGCTCAGT**	/
	R	GTATCGCGTCTAGGTTCGAAC	/
	P	A**GGTCCGAGCC**	5’FAM, 3’BHQ1
hcmv-miR-UL22A-5p	Capture probe	**GAAGGCTAGTTA**	3’-biotin-TEG
	RT	TTGAGGTAAATCCTTGGTCGATGGAGTCTTTACCTCAA**T[LNA-C] TCAC**	LNA
	F	CATCCATACGAA**TAA[LNA-C] TAGCCTTC**	LNA
	R	TAAATCCTTGGTCGATGGAGTC	/
	P	TCAA**TCTCACGGG**	5’FAM, 3’BHQ1
hcmv-miR-	Capture	**CACACCTATCATA**	3’-biotin-TEG

US5-2-3p	probe		
	RT	CTCTCAAGAGGATCTAAGCTGTGGTCGGTTCTTG AGAGA**AGACAT**	/
	F	ACACGCGAAGT**TATGATAGGTG**	/
	R	GGATCTAAGCTGTGGTCGGT	/
	P	AGAGA**ACATCGTCAC**	5'-BHQ1, 3'-FAM
hcmv-miR-US4-3p	Capture probe	**TAGCGG[LNA-G] CTGTCA**	3’-biotin-TEG
	RT	CTCAACTGTATCGCGTCTAGGTTCGAACTTCAGT TGAG**AGAGGT**	/
	F	ATTATACCAT**TGACAGCCCG**	/
	R	GTATCGCGTCTAGGTTCGAAC	/
	P	TTGAG**AGAGGTGTAG**	5’FAM, 3’BHQ1
hcmv-miR-US25-2-3p	Capture probe	**GCTCTCCAAGTGGAT**	3’-biotin-TEG
	RT	TTGAGGTATATCGCGTCTAGGTTCGAACTTTACC TCAA**ACCGCG**	/
	F	CCACGATA**ATCCACTTGGAGA**	/
	R	GTATATCGCGTCTAGGTTCGAAC	/
	P	**ACCGCGGGAGC**	5’FAM, 3’BHQ1
ebv-miR-BART7-3p	Capture probe	**ACTGGACTATGATG**	3’-biotin-TEG
	RT	GATGCACATCTTGAGTCTAGCATCGAGCATTGTG	/

		CATC**CCCTG**	
	F	ACATCGG**CATCATAGTCCAGT**	/
	R	TCTTGAGTCTAGCATCGAGCAT	/
	P	ATCCCCTG**GACA**	5’FAM, 3’BHQ1
ebv-miR-BART2-5p	Capture probe	G[2`-methoxythyl-A]A[2`-methoxythyl-T]GC[LNA-A]GAAAATA	Methoxythyl, LNA, 3’-biotin-TEG
	RT	GATAAACGTGCGACAGATCCTAGACAGCGACGTTTATC**GCAAGG**	/
	F	TCTCCTGACTC**TATTTTCTGCAT**	/
	R	GCGACAGATCCTAGACAGCG	/
	P	AGCAAGG**GCGAA**	5’FAM, 3’BHQ1
ebv-miR-BART3-3p	Capture probe	**ACTAGTGGTGCG**	3’-biotin-TEG
	RT	CTCATTGGCGATCCTTGGTCGATGGTGAAGCCAATGAGA**CACCTG**	/
	F	TCTCTAGCAG**CGCACCACTA**	/
	R	GATCCTTGGTCGATGGTGAA	/
	P	CACCTG**GTGACT**	5’FAM, 3’BHQ1

TEG: triethylene glycol. “/” denotes unmodified nucleotides. LNA: locked nucleic acid modification. FAM: carboxyfluorescein. BHQ1: black hole quencher 1. bolded bases represent sequences complementary or concordant with miRNA.

Supplementary Table 8 Characteristics of clinical samples for colorectal cancer diagnosis

	Age (years) range	Age (years) median	Sex (n, %) Man	Sex (n, %) Female	Tumor location (n, %)	Sample type
Colorectal cancer patients	32-80	59.5	31 (64.6)	17 (35.4)	Colon: 27 (56.3)<br>Rectum: 21 (43.7)	Serum
Healthy person	39-75	64	28 (58.3)	23 (41.7)	/	Serum

"/" means not applicable.

Supplementary Table 9 Demographic and clinical characteristics of nasopharyngeal carcinoma (NPC) patients and healthy controls (HC)

	Age (years) range	Age (years) median	Sex (n, %) Man	Sex (n, %) Female	Clinical stage (n, %)	Sample type
NPC	17-65	48.0	31 (77.50)	9 (22.50)	III: 4 (10)<br>IVB: 36 (90)	Plasma
HC	18-76	49.0	31 (77.50)	9 (22.50)	/	Plasma

"/" means not applicable.

Supplementary Table 10 Analysis of miRNA characteristics included in this study

	miRNA	Sequence	Length (nt)	GC%	Tm	ΔG (Kcal/mol)	Loop Tm	Loop ΔG (Kcal/mol)
Human	hsa-miR-92a-3p	UAUUGCACUUGUCCCG GCCUGU	22	54.55%	73.6	-44.8	/	1.2
	hsa-miR-320a	AAAAGCUGGGUUGAGA GGGCGA	22	54.55%	74.4	-46.1	/	1.8
	hsa-miR-19a-3p	UGUGCAAAUCUAUGCA AAACUGA	23	34.78%	66.2	-40.1	77	-2.6
	hsa-miR-423-5p	UGAGGGGCAGAGA GCGAGACUUU	24	54.17%	75.1	-47.5	7	0.9
HCMV	hcmv-miR-US25-1-5p	AACCGCUCAGUGGCUC GGACC	21	66.67%	75.4	-45.0	67	-2.4
	hcmv-miR-UL22A-5p	UAACUAGCCUUCCCGU GAGA	20	50.00%	62.3	-37.5	/	2.4
	hcmv-miR-US5-2-3p	UAUGAUAGGUGUGACG AUGUCU	22	40.91%	59.2	-35.6	/	2.3

	hcmv-miR-US4-3p	UGACAGCCCGCUACAC CUCU	20	60.00%	67.6	-39.3	/	2.6
	hcmv-miR-US25-2-3p	AUCCACUUGGAGAGCU CCCGCGGU	24	62.5%	80.3	-51.5	27	-0.2
	ebv-miR-BART3-3p	CGCACCACUAGUCACC AGGUGU	22	59.09%	69.4	-40.7	56	-1.8
EBV	ebv-miR-BART7-3p	CAUCAUAGUCCAGUGU CCAGGG	22	54.55%	66.4	-39.4	/	1.0
	ebv-miR-BART2-5p	UAUUUUCUGCAUUCGC CCUUGC	22	45.45%	71.6	-44.7	49	-0.9

nt: nucleotides. "Tm" means melting temperature. ΔG: Gibbs free-energy change. "/" means not applicable. "HCMV" means Human Cytomegalovirus. "EBV" means Epstein–Barr virus. Tm, ΔG, Loop Tm and Loop ΔG are calculated using Oligo 6 software.
