# Supplementary material for: A selective enrichment and specific probe terminal mediated strategy for highly sensitive detection of microRNAs
Source: Nat Commun. 2026 Mar 16;17:4377. doi: 10.1038/s41467-026-70811-7 (PMC13184116; doi:10.1038/s41467-026-70811-7)
Supplement: Supplementary file 2 — Summary [file 41467_2026_70811_MOESM2_ESM.pdf]

nature portfolio

Corresponding author(s): Shiyin Zhang

Last updated by author(s): Feb 26, 2026

Reporting Summary

Nature Portfolio wishes to improve the reproducibility of the work that we publish. This form provides structure for consistency and transparency in reporting. For further information on Nature Portfolio policies, see our Editorial Policies and the Editorial Policy Checklist.

Statistics

For all statistical analyses, confirm that the following items are present in the figure legend, table legend, main text, or Methods section.

n/a	Confirmed	
☐	☒	The exact sample size (*n*) for each experimental group/condition, given as a discrete number and unit of measurement
☐	☒	A statement on whether measurements were taken from distinct samples or whether the same sample was measured repeatedly
☐	☒	The statistical test(s) used AND whether they are one- or two-sided<br>*Only common tests should be described solely by name; describe more complex techniques in the Methods section.*
☒	☐	A description of all covariates tested
☐	☒	A description of any assumptions or corrections, such as tests of normality and adjustment for multiple comparisons
☐	☒	A full description of the statistical parameters including central tendency (e.g. means) or other basic estimates (e.g. regression coefficient) AND variation (e.g. standard deviation) or associated estimates of uncertainty (e.g. confidence intervals)
☐	☒	For null hypothesis testing, the test statistic (e.g. *F*, *t*, *r*) with confidence intervals, effect sizes, degrees of freedom and *P* value noted<br>*Give P values as exact values whenever suitable.*
☒	☐	For Bayesian analysis, information on the choice of priors and Markov chain Monte Carlo settings
☒	☐	For hierarchical and complex designs, identification of the appropriate level for tests and full reporting of outcomes
☐	☒	Estimates of effect sizes (e.g. Cohen's *d*, Pearson's *r*), indicating how they were calculated

Our web collection on statistics for biologists contains articles on many of the points above.

Software and code

Policy information about availability of computer code

Data collection	The real-time fluorescence data for the SE-SPTM-PCR method were collected using commercial real-time PCR machine software (Bio-Rad CFX 96). The real-time fluorescence data for conventional miRNA detection methods were collected using commercial real-time PCR machine software (ABI 7500). The Sequencing data for non-specific amplification products and HCMV miRNA expression were collected using Illumina platform (Novaseq 6000).
Data analysis	Mean was calculated with standard formulas in Excel (Office 365). Histograms and scatterplots were created using Prism (v10.1.2). Chi-square tests, correlation analyses and two-tailed Mann-Whitney U test were conducted using GraphPad Prism 10. ROC curve analyses were performed using SPSS 27.

For manuscripts utilizing custom algorithms or software that are central to the research but not yet described in published literature, software must be made available to editors and reviewers. We strongly encourage code deposition in a community repository (e.g. GitHub). See the Nature Portfolio guidelines for submitting code & software for further information.

Data

Policy information about availability of data

All manuscripts must include a data availability statement. This statement should provide the following information, where applicable:

- Accession codes, unique identifiers, or web links for publicly available datasets
- A description of any restrictions on data availability
- For clinical datasets or third party data, please ensure that the statement adheres to our policy

The data generated in this study are provided in the main text and Supplementary Information. Source data are provided as a Source Data file.

Research involving human participants, their data, or biological material

Policy information about studies with human participants or human data. See also policy information about sex, gender (identity/presentation), and sexual orientation and race, ethnicity and racism.

Reporting on sex and gender	Human serum and plasma samples, from males and females, were randomly pulled out from the biorepository.
Reporting on race, ethnicity, or other socially relevant groupings	The study did not report on racial, ethnic, or other socially relevant groupings.
Population characteristics	Serum samples from 48 colorectal cancer patients, plasma samples from 32 hematopoietic stem cell transplantation patients, 40 nasopharyngeal carcinoma patients, and related healthy individuals were obtained from the biorepository. Detailed population information is provided in Supplementary Table S8 and S9.
Recruitment	No participants were recruited for the study.
Ethics oversight	This study was approved by the Ethics Committee of Xiamen University and Fujian Medical University Union Hospital.

Note that full information on the approval of the study protocol must also be provided in the manuscript.

Field-specific reporting

Please select the one below that is the best fit for your research. If you are not sure, read the appropriate sections before making your selection.

☒ Life sciences ☐ Behavioural & social sciences ☐ Ecological, evolutionary & environmental sciences

For a reference copy of the document with all sections, see nature.com/documents/nr-reporting-summary-flat.pdf

Life sciences study design

All studies must disclose on these points even when the disclosure is negative.

Sample size	We utilized plasma samples from 48 colorectal cancer patients, 32 hematopoietic stem cell transplantation patients, 40 nasopharyngeal carcinoma patients, and a number of healthy individuals.
Data exclusions	No data were excluded from the analyses.
Replication	All the experiments were conducted at least in duplicate, and all attempts at replication were successful.
Randomization	Plasma samples were randomly selected from the biorepository.
Blinding	The analytical studies were not carried out blinded but included biological replicates to ensure minimal experimental bias.

Reporting for specific materials, systems and methods

We require information from authors about some types of materials, experimental systems and methods used in many studies. Here, indicate whether each material, system or method listed is relevant to your study. If you are not sure if a list item applies to your research, read the appropriate section before selecting a response.

Materials & experimental systems

n/a	Involved in the study
☒	☐ Antibodies
☐	☒ Eukaryotic cell lines
☒	☐ Palaeontology and archaeology
☒	☐ Animals and other organisms
☒	☐ Clinical data
☒	☐ Dual use research of concern
☒	☐ Plants

Methods

n/a	Involved in the study
☒	☐ ChIP-seq
☒	☐ Flow cytometry
☒	☐ MRI-based neuroimaging

Eukaryotic cell lines

Policy information about cell lines and Sex and Gender in Research

Cell line source(s)	MRC5 (ATCC, cat # CCL-171)
Authentication	The cell line has not been authenticated. It was obtained commercially from trusted suppliers.
Mycoplasma contamination	The cell line tested negative for mycoplasma contamination.
Commonly misidentified lines (See ICLAC register)	No commonly misidentified cell line was used.

Plants

Seed stocks	*Report on the source of all seed stocks or other plant material used. If applicable, state the seed stock centre and catalogue number. If plant specimens were collected from the field, describe the collection location, date and sampling procedures.*
Novel plant genotypes	*Describe the methods by which all novel plant genotypes were produced. This includes those generated by transgenic approaches, gene editing, chemical/radiation-based mutagenesis and hybridization. For transgenic lines, describe the transformation method, the number of independent lines analyzed and the generation upon which experiments were performed. For gene-edited lines, describe the editor used, the endogenous sequence targeted for editing, the targeting guide RNA sequence (if applicable) and how the editor was applied.*
Authentication	*Describe any authentication procedures for each seed stock used or novel genotype generated. Describe any experiments used to assess the effect of a mutation and, where applicable, how potential secondary effects (e.g. second site T-DNA insertions, mosiacism, off-target gene editing) were examined.*
